# The anti-inflammatory effect of myrrh ethanolic extract in comparison with prednisolone on an autoimmune disease rat model induced by silicate

**DOI:** 10.1007/s10787-022-01042-7

**Published:** 2022-08-05

**Authors:** Dina E. ElMosbah, Marwa S. Khattab, Shimaa R. Emam, Hala M. F. El Miniawy

**Affiliations:** 1 Department of Pathology, Faculty of Veterinary Medicine, Cairo University, Giza, Egypt; 2 Department of Pharmacology, Faculty of Veterinary Medicine, Cairo University, Giza, 12211 Egypt

**Keywords:** Autoimmune diseases, Prednisolone, Myrrh, Histopathology, TNF-α, Glomerulonephritis

## Abstract

Autoimmune disease is a complex chronic disease that triggers immune activation against autoantigens resulting in tissue damage. Epidemiological data showed that autoimmune diseases are increasing worldwide over the last decades owing to increased environmental pollution. This study investigates the therapeutic effect of myrrh as a natural medicine compared to prednisolone in the treatment of immune-mediated glomerulonephritis induced by silicate. The autoimmune disease model in rats was induced by injecting 5 mg crystalline sodium silicate suspension subcutaneously once weekly for 20 weeks, and then the rats were treated either with myrrh extract or prednisolone or with both for 6 weeks. Liver and kidney function tests, histopathology, and immunohistochemistry of TNF-α expression in kidney tissue were performed. The creatinine significantly elevated in silica-treated group and decreased in other treated groups. Histopathology of the kidney revealed improvement of glomerular and tubular basement thickness in all treated groups, but the inflammatory cell count slightly decreased in the group treated with myrrh than the other treated groups which showed a marked decrease. TNF-α expression was significantly decreased in all treated groups. Interestingly, the myrrh did not produce hepatic lesions and improve the side effect of prednisolone in the liver when taken in combination. Therefore, myrrh extract possessed anti-inflammatory properties and counteracted the side effect of prednisolone on the liver. Myrrh extract can serve as a conjunctive therapy with prednisolone to treat autoimmune diseases.

## Introduction

Autoimmunity is an immune response against self-antigens leading to demonstrable pathology (Pollard et al. 2010). Epidemiological studies indicated that autoimmune diseases (ADs) are the 10th most common cause of mortality in developing countries (Nielsen and Hultman. 2002). AD initiation is due to multifactorial reasons including genetic, hormonal, environmental, and immunological factors. Extrinsic factors such as drugs, chemicals, microbes, and environmental constituents including silica, mercury, cadmium, gold, and L canavanine can trigger the initiation of an autoimmune response (Hess. 2002; Pollard et al. 2010). The mechanism by which these substances induce autoimmunity can be grouped into three general categories: inhibiting deletion of newly generated autoreactive cells permitting their release to the circulation, modification of gene expression in the immune cells allowing lymphocytes to react to signals normally not enough to initiate a response or allowing the antigen-presenting cells to abnormally trigger a response and activate autoreactive cells, and alteration of self-antigen such that they are recognized by the immune system as foreign (Rao and Richardson. 1999).

Suppression of the pathologic autoimmune response is the main therapeutic option for autoimmune disorders. This involves the use of immunosuppressive and immunomodulatory therapy targeting cytokines and immune cells crucial to the inflammatory process besides the use of symptomatic therapy (Singer et al. 1995; Dalakas. 2006).

Treatment options include medications such as nonsteroidal anti-inflammatory drugs, biological agents, and glucocorticoids. Corticosteroids especially systemic ones such as prednisolone are one of the most commonly prescribed drugs in autoimmune diseases due to their rapid and broad-spectrum actions on immune cells (van der Goes et al. 2014; Hillier. 2007). In vivo studies confirmed the anti-inflammatory property of prednisolone in an induced SLE model in female NZB/W mice (Chafin et al. 2013). Nevertheless, corticosteroids have many side effects including osteoporosis, glaucoma, and metabolic abnormalities such as diabetes and hypercholesterolemia (van der Goes et al. 2014). Also, corticosteroid long therapy can cause hepatic enlargement, hepatic steatosis, centrilobular ballooning degeneration, and Mallory bodies (Candelli et al. 2003).

Natural agents are now gaining more attention regarding their use in disease therapy since they are a safe and cheap source of a drug (Astry et al. 2015). Myrrh, present in the stem of Commiphora molmol, was reported to have anti-microbial, analgesic, anti-oxidant, and anti-inflammatory properties in animal studies (Shalaby and Hammouda. 2014; Rosenthal et al. 2017). It has been used in traditional medicines to treat variable diseases Pećanac et al. 2013). Myrrh resin was used previously in cases of arthritis, tumors, trauma, and fractures (Shen et al. 2012). U.S Food and Drug Administration approved the safety of myrrh. Therefore, the current study was performed to investigate the anti-inflammatory effect of myrrh on an autoimmune disease model induced by silica in comparison with the conventional treatment (Prednisolone).

## Material and methods

### Animals used

Adult mature female Albino rats weighing from 200-250 g were used in this study. Rats were purchased from Vacsera Laboratory Animal House, Egypt. Animals were reared in cages at 25±2 °C, a 12/12 h light-dark cycle, and on sawdust bedding. Rats were fed on rat pellets composed of 10% wheat bran, 44% soybean powder, 22% net protein, 4.7% fats, 3.3% fibers, fish meal, molasses, salts (sodium chloride, calcium carbonate, and calcium phosphate), and methionine (Cairo Agriculture Development Company, 6th October City, Egypt). Water was provided to rats ad libitum. All procedures were approved (vetCu1022019064) and performed according to the guidelines of the Institutional Animal Care and Use Committee, Faculty of Veterinary Medicine, Cairo University. The experiment was carried out in the Pathology Department, Faculty of Veterinary Medicine, Cairo University. Euthanasia was performed by cervical dislocation under tranquilization (2% isoflurane by inhalation).

### Chemicals

Sodium silicate (Na2SiO3) was purchased from LOBA Chemie, Mumbai, India, and was used as a suspension in a dose (5mg of crystalline sodium silicate in 0.2mL of 0.9% saline). Prednisolone was purchased from SANOFI Company, Egypt.

### Myrrh ethanolic extract

Myrrh was purchased from a local market of medicinal plants and herbs in Cairo, Egypt. The myrrh resin was finely grounded and kept in a tightly closed glass container at room temperature pending phytochemical investigation. To each 250 *g* of the resin powder, one liter of 70% ethyl alcohol was added to a wide-mouth plastic container and kept overnight. On the next morning, the container content was filtered using double-layer gauze. The ethanol in the filtrate was then evaporated in a rotatory evaporator at a temperature of 40 °C under reduced pressure using a vacuum pump. Each 1kg from myrrh yielded 32 *g* of crude extract. 10% of myrrh extract was dissolved in distilled water using a few drops of Tween 80 as a suspending agent. The selected dose of myrrh extract was calculated according to the LD50 of resin extract. The table described by Paget and Barnes (1962) was used to determine the doses of standard drugs used for laboratory animal experiments.

### Experimental design

Forty female rats were assigned into eight groups. **Group A** was injected subcutaneously with 0.2 mL of 0.9% normal saline and served as control. **Group B (Prednisolone group)** received 10mg/kg of prednisolone daily by oral gavage for 6 weeks. **Group C (Myrrh group)** received 500mg/kg of myrrh extract daily by oral gavage for 6 weeks. **Group D (Prednisolone/Myrrh group)** received a combination therapy of 10mg/kg of prednisolone and 500mg/kg of myrrh extract daily by oral gavage for 6 weeks. **Group E** –**Group G** were injected subcutaneously with 0.2 mL of 5mg crystalline sodium silicate suspension once weekly for 20 weeks to induce autoimmune disease then, **Group F (Silicate/Prednisolone group) was** treated with 10mg/kg of prednisolone daily by oral gavage for 6 weeks, **Group G (Silicate/Myrrh group)** treated with 500mg/kg of myrrh extract daily by oral gavage for 6 weeks, and **Group H (Silicate/Prednisolone/Myrrh group)** treated with combination therapy of 10mg/kg of prednisolone and 500mg/kg of myrrh extract daily by oral gavage for 6 weeks. Samples were collected at 3 and 6 weeks post-treatment. Rats were euthanized at the end of the experiment by cervical dislocation.

### Determination of biochemical tests

Alanine aminotransferase (ALT), aspartate aminotransferase (AST), urea, and creatinine were measured in the serum of rats in all groups using reagent test kits (Spectrum Diagnostics, Cairo, Egypt) according to the manufacturer’s protocol.

### Histopathological examination

The kidney and liver specimens were fixed in 10% neutral buffered formalin and processed by paraffin embedding technique. The paraffin-embedded tissues were sectioned (3-4 µm thick) using a microtome (Leica 2135, Germany), and stained with hematoxylin and eosin stain and special stains such as periodic acid Schiff (PAS) (Bancroft and Gamble. 2013). Microscopic examination was carried out using a light microscope (BX50F4, Olympus, Japan).

Microscopic grading of renal damage was performed according to the following parameters: glomerular hypercellularity, crescent formation, glomerular basement membrane (GBM) thickening of 50 glomeruli/rat, and tubular basement membrane thickening. The above parameters were semiquantitatively assessed and scored according to the following scale: (0) absent; (1) weak < 25%; (2) mild 25–50%; (3) moderate 50–75%; (4) severe > 75% of the tissues affected according to Heeringa et al. (1996). Other lesions such as tubular luminal cells and cast and tubular degeneration were recorded as present or absent. Interstitial inflammatory cell infiltration was scored according to Wu et al. (2010) which were assessed as follows: score 0, foci with < 5 infiltrated mononuclear cells (single or multiple foci) or < 5 cells per high-power field (diffuse changes); score 1, foci with < 25 infiltrated mononuclear cells (single or multiple foci) or < 25 cells HPF (diffuse changes); score 2, foci with 25–50 infiltrated mononuclear cells (single or multiple foci) or 25-50 cells per HPF (diffuse changes); score 3, foci with 50–75 infiltrated mononuclear cells (single or multiple foci) or 50–75 cells per HPF (diffuse changes); score 4, foci with > 75 infiltrated mononuclear cells (single or multiple foci) or > 75 cells per HPF (diffuse changes).

### Immunohistochemistry (IHC)

The expression of TNFα (tumor necrosis factor-alpha) in kidneys was evaluated in paraffin tissue sections of all groups using the immunoperoxidase technique as mentioned by Hsu et al. (1981) Briefly, tissue sections were deparaffinized, rehydrated, and underwent antigen retrieval. They were then incubated with 1:100 TNFα primary antibody (Santa Cruz, USA) followed by the secondary biotinylated antibody, and later by the avidin peroxidase complex (Vactastain ABC peroxidase kit, Vector Laboratories, Burlingame, CA, USA). Color development was performed by chromogen 3, 3-diaminobenzidine tetrahydrochloride (DAB, Sigma Chemicals, Perth, Australia). Slides were counterstained with hematoxylin and then examined under a light microscope. TNFα expression in kidney sections was quantified in 10 fields at a magnification power of 200X/rat using Image J software. Morphometric results were expressed as mean ± SEM of the positive area percent.

### Statistical analysis

Statistical analysis was carried out using the statistical package SPSS, version 8.0 (SPSS Inc., Chicago, IL, U.S.A.). Parametric data were analyzed by the Anova test, and nonparametric data (lesion score) were as analyzed by the Kruska–Wallis test. The post hoc test (Mann–Whitney test) was performed to detect significance among groups. P values less than 0.05 were considered significant.

## Results

### Biochemical analysis

At 3 weeks post-treatment**,** there was a significant increase in serum levels of ALT and AST in group B (Prednisolone group), group E (Silicate group), and group F (Silicate/Prednisolone group), respectively, compared to the other groups with marked significant in serum levels of ALT in group E. The creatinine and urea recorded a significant decrease in group H (Silicate/Prednisolone/Myrrh group), G (Silicate/Myrrh group), and F, respectively, than group E (Table 1).

**Table 1:** Liver and kidney function tests in different groups at 3 weeks post-treatment

Test at 3wks	Group A	Group B	Group C	Group D	Group E	Group F	Group G	Group H
ALT (IU/L)	21 ± 1.4^**a**^	41.3 ± 4.6^**d,e**^	23 ± 2.5^**a**^	30.3 ± 2.3^**b,c**^	48 ± 1.5^**e**^	34.5 ± 1.2^**c,d**^	21.9 ± 3.1^**a**^	27.3 ± 2.1^**a,b,c**^
AST (IU/L)	105 ± 1.4^**a**^	137.6 ± 5.5^**b**^	106.8 ± 2.9^**a**^	113 ± 1.5^**a**^	139.6 ± 3.8^**b**^	153.6 ± 9.6^**b**^	105 ± 11.5^**a**^	113 ± 10.3^**a**^
BUN (mg/dl)	21.6 ± 2.1^**a,b**^	20 ± 0.5^**a**^	20.5 ± 1.2^**a,b**^	22.6 ± 1.8^**a,b**^	44.5 ± 0.8^**c**^	26.6 ± 1.4^**b**^	24.6 ± 4.1^**a,b**^	20.1 ± 0.7^**a,b**^
Creatinine (mg/dl)	**1.2 ± 0.7**^**a,b**^	**1.2 ± 0.08**^**a,b**^	**1.1 ± 0.03**^**a,b**^	**1.1 ± 0.03**^**a**^	**1.8 ± 0.07**^**c**^	**1.3 ± 0.03**^**b**^	**1.2 ± 0.03**^**a,b**^	**1.2 ± 0.05**^**a,b**^

All data presented as mean ± SE. Values bearing different superscripts (a,b,c,d,e) are significant at P values less than 0.05

At 6 weeks post-treatment, the concentrations of ALT in serum recorded their highest value in group F (60 IU/L) while it showed a significant decrease in group G and H (26.6 and 29.2 IU/L) respectively. The creatinine still showed decreased values in groups H, G, and F (1, 1.05, 1.1 mg/dl) than in group E (1.8 mg/dl) (Table 2).

**Table 2:** Liver and kidney function tests in different groups at 6 weeks post-treatment

Test at 3wks	Group A	Group B	Group C	Group D	Group E	Group E	Group G	Group H
ALT (IU/L)	19 ± 0.5^**a**^	40.3 ± 2.6^**c**^	14.8 ± 2.4^**a**^	27.3 ± 1.2^**b**^	46.3 ± 1.7^**C**^	60 ± 3.7^**d**^	26.6 ± 2.02^**a**^	29.2 ± 2.2^b^
AST (IU/L)	75.6 ± 5.8^**a**^	150 ± 8.6^**d**^	79 ± 4.9^**a**^	131 ± 3.4^**b,c**^	148.3 ± 0.8^**d**^	142.3 ± 3.9^**c,d**^	120 ± 2.02^**b**^	122.7 ± 1.5^**b**^
BUN (mg/dl)	19 ± 0.5^a^	19.5 ± 0.2^**a**^	19.2 ± 0.26^**a**^	19 ± 0.5^**a**^	36.6 ± 1.6^**c**^	21.6 ± 1.2^**a,b**^	21 ± 2^**a,b**^	24.3 ± 0.8^**b**^
Creatinine (mg/dl)	**0.7 ± 0.6**^**a**^	**0.6 ± 0.03**^**a**^	**0.9 ± 0.03**^**b**^	**1 ± 0.04**^**b**^	**1.8 ± 0.3**^**c**^	**1.1 ± 0.5**^**b**^	**1.05 ± 0.2**^**b**^	**1 ± 0.01**^**b**^

All data presented as mean ± SE. Values bearing different superscripts (a,b,c,d) are significant at P values less than 0.05

### Histopathology

The microscopic examination of the kidney and liver sections of the control group (A) showed normal histological structures. NO remarkable changes were observed in group B (Prednisolone group), group C (Myrrh group), and group D (Prednisolone/Myrrh group) in kidney tissue.

### Kidney

**Fig. 1:**
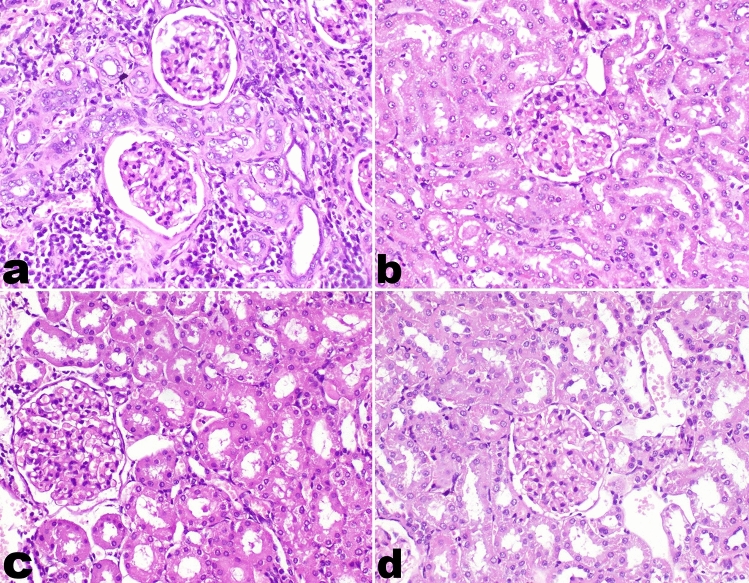
Histopathology of kidneys of rats. **a** Thickening of glomerular basement membrane with marked periglomerular interstitial inflammatory cells infiltration and fibrosis in group E (Silicate group), **b** there is no inflammatory reaction observed in group F (Silicate/Prednisolone group), **c** mild mononuclear inflammatory cells infiltration in group G (Silicate/Myrrh group), **d** no inflammatory reaction observed in group H (Silicate/ Prednisolone/Myrrh group) (20X). H and E stain

**Fig. 2:**
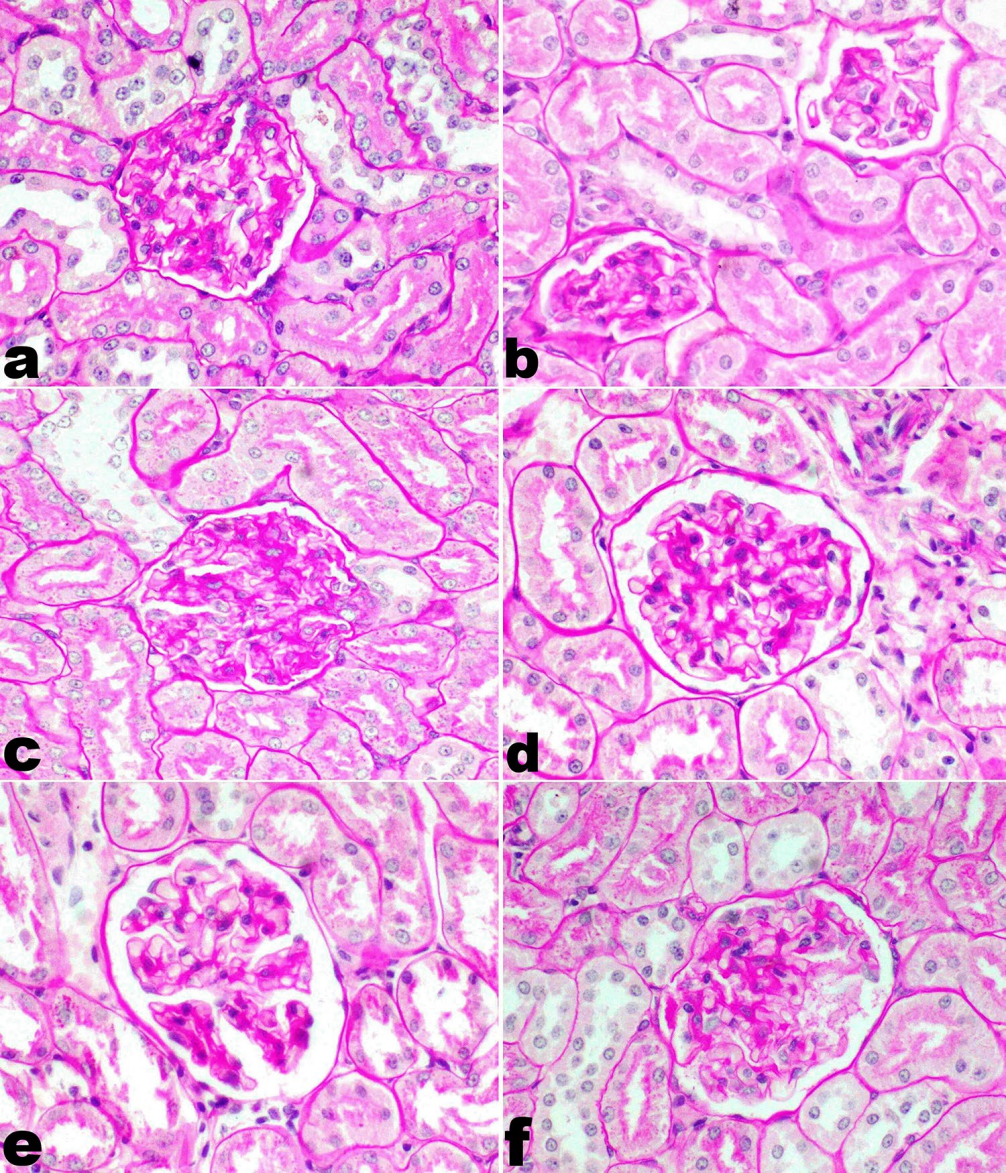
Periodic acid–Schiff staining of kidney tissue. **a** Normal glomerular and tubular basement membrane thickness in the control group (A), **b** severe PAS-positive thickening of the glomerular capillary tuft and basement membrane in group E (Silicate group), **c** group F (Silicate/Prednisolone group) similar to group A, **d**, **e** mild thickening in group G (Silicate/Myrrh group), **f** and also mild thickening in group H (Silicate/Prednisolone/Myrrh group) (40X)

The immune-mediated glomerulonephritis induced by silica was manifested by the thickening of the glomerular capillary tuft and tubular basement membrane. Many glomeruli showed foci of mesangial proliferation. Mononuclear inflammatory cell infiltration was significantly observed in focal areas in the interstitial tissue and perivascular area with the proliferation of fibrous connective tissue in the interstitial tissue. Furthermore, renal tubules suffered from mild to moderate epithelial degeneration and renal casts. On the other hand, the kidney in group F (Silicate/Prednisolone group) and group H (Silicate/Prednisolone/Myrrh group) showed mild thickening in the glomerular basement membrane without inflammatory reaction. In group G (Silicate/Myrrh group), there was moderate thickening in the glomerular basement membrane with mild inflammatory cell infiltration (Fig. 1). The thickening of the glomerular capillary tuft and tubular basement membrane was demonstrated by PAS stain in all experimental groups (Fig. 2).

### Renal lesion score

**Fig. 3:**
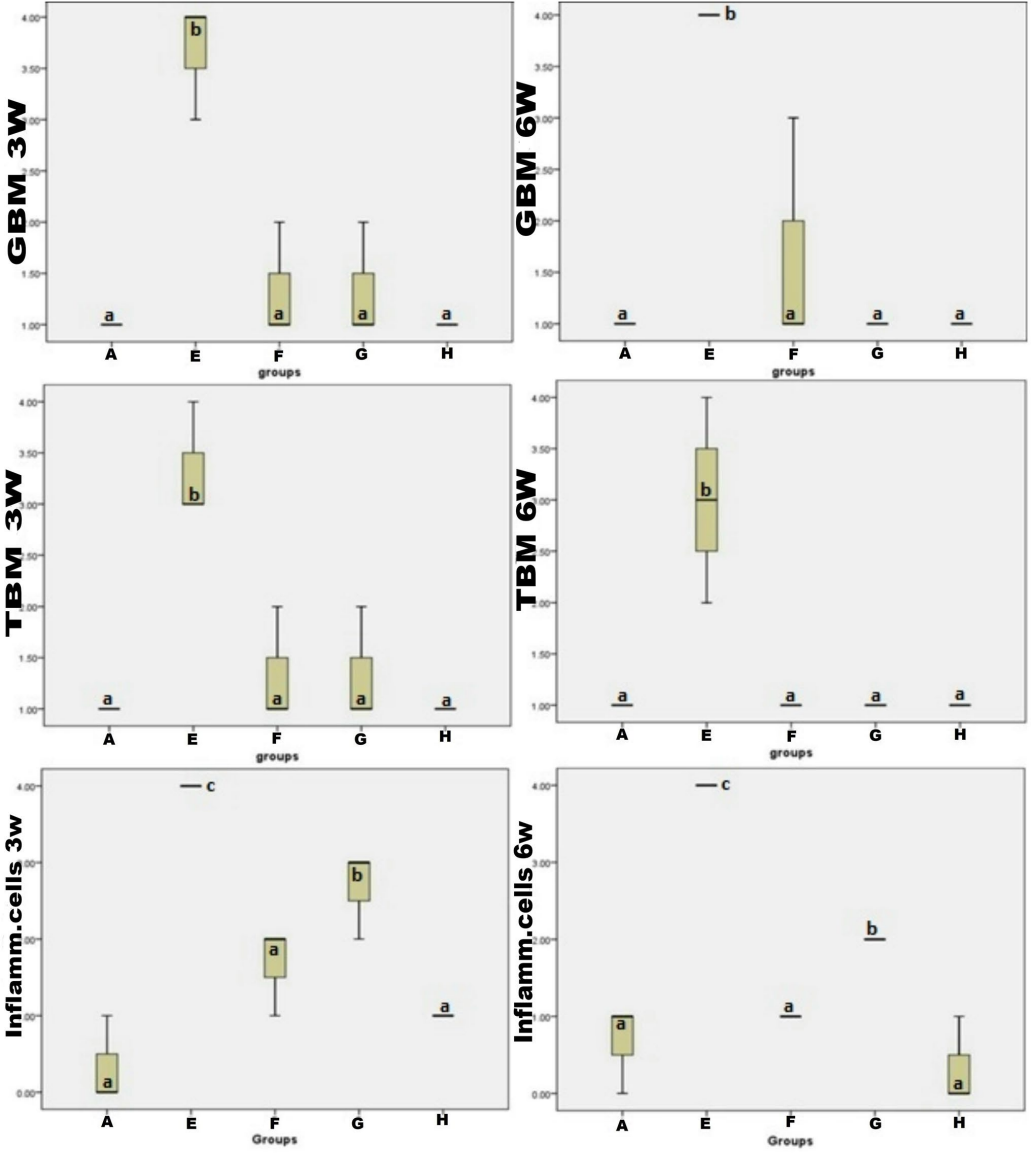
Box plots of GBM, TBM, and inflammatory cells infiltration lesion scores in renal tissue at 3wks and 6wks post-treatment in different groups. The boxes are the interquartile range (IQR). The medians are the thick middle lines. The maximum and minimum values are represented by the thin horizontal lines at the top and bottom. Boxes bearing different superscripts (a, b, c) are significant at P values less than 0.05

At 3wks and 6wks post-treatment, group E (Silicate group) showed a significant increase in GBM and TBM scores compared to the other treated groups and control group. While these score lesions decreased in group H (Silicate/Prednisolone/Myrrh group), G (Silicate/Myrrh group), and F (Silicate/Prednisolone group), respectively. The inflammatory cell count score was significantly decreased in group H and F compared to group E, but it slightly decreased in group G than in group E. There was no significant difference between group F, group H, and the control group at 3wks and 6wks post-treatment. No significant difference was recorded between-group F and group G at 3wks. While at 6wks post-treatment, group F significantly decreased than group G (Fig. 3).

### Immunohistochemistry of TNF-α in the kidney:

**Fig. 4:**
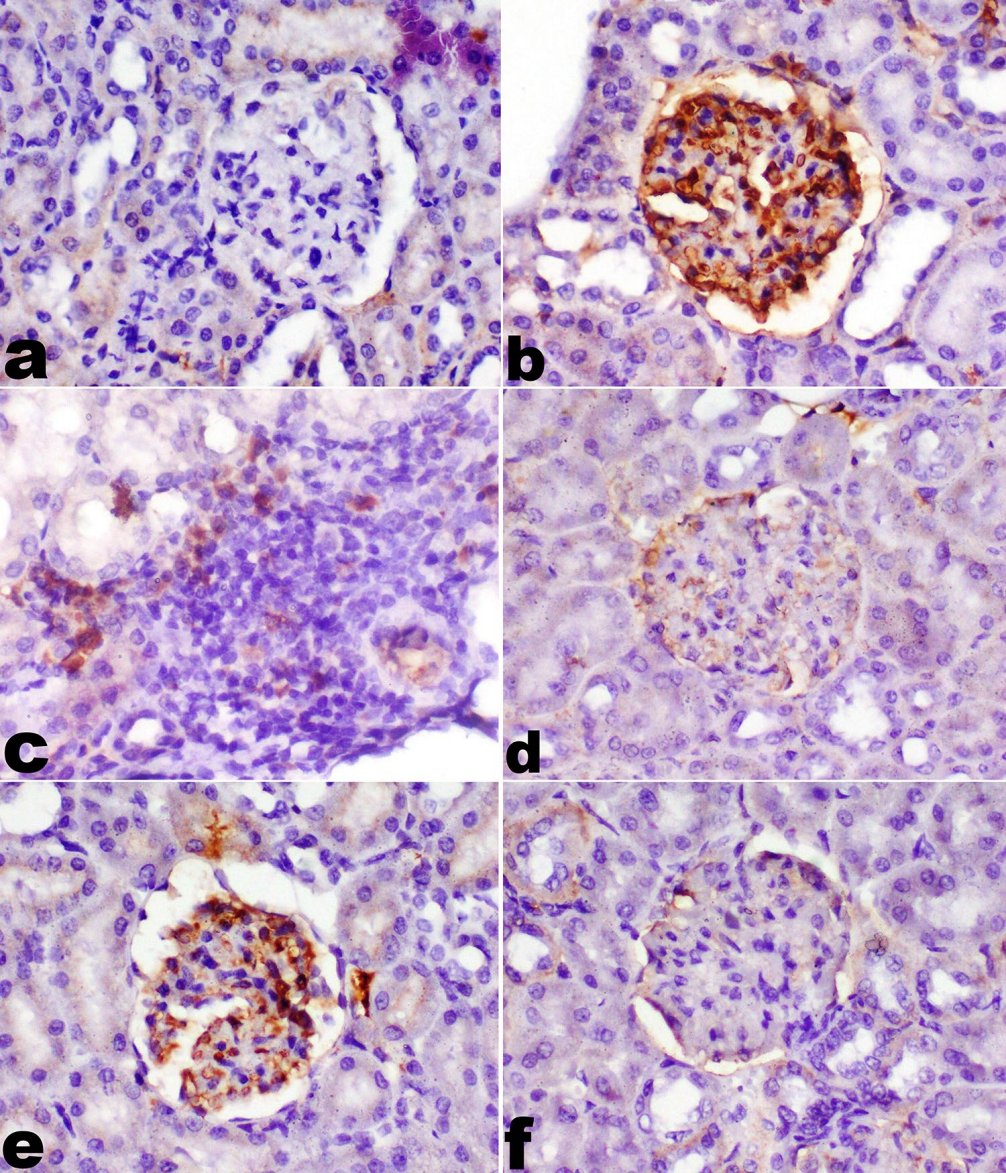
Immunohistochemistry of TNF-α expression in renal tissue in different groups. **a** No TNF-α expression in the control group (A), **b** marked expression of TNF-α in glomerular parietal epithelial cells and mesangial cells in group E (Silicate group), **c** expression of TNF-α in mononuclear inflammatory cells, **d** no TNF-α expression in group F (Silicate/Prednisolone group), **e** mild TNF-α expression in glomerular parietal epithelial cells in group G (Silicate/Myrrh group) **f** no TNF-α expression in group H (Silicate/Prednisolone/Myrrh group) (40X)

**Fig. 5:**
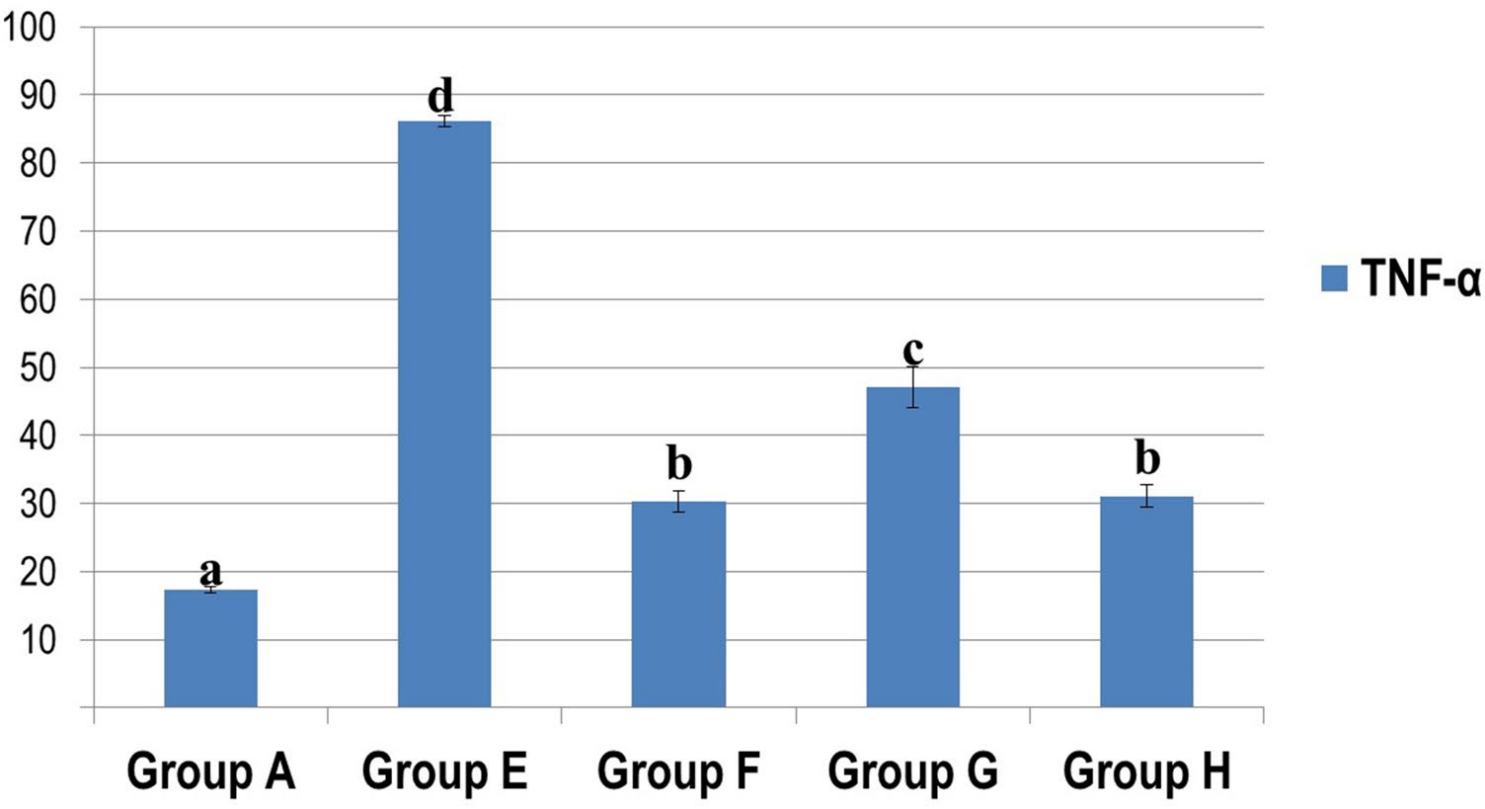
The area % of TNF-α expression in the kidney

TNF-α expression was significantly decreased in group G (Silicate/Myrrh group) compared to group E (Silicate group), while the least expression of TNF-α was recorded in group F (Silicate/Prednisolone group) and H (Silicate/Prednisolone/Myrrh group) (Fig. 4) and as shown in the chart of area% of TNF-α expression (Fig. 5).

### Liver

**Fig. 6:**
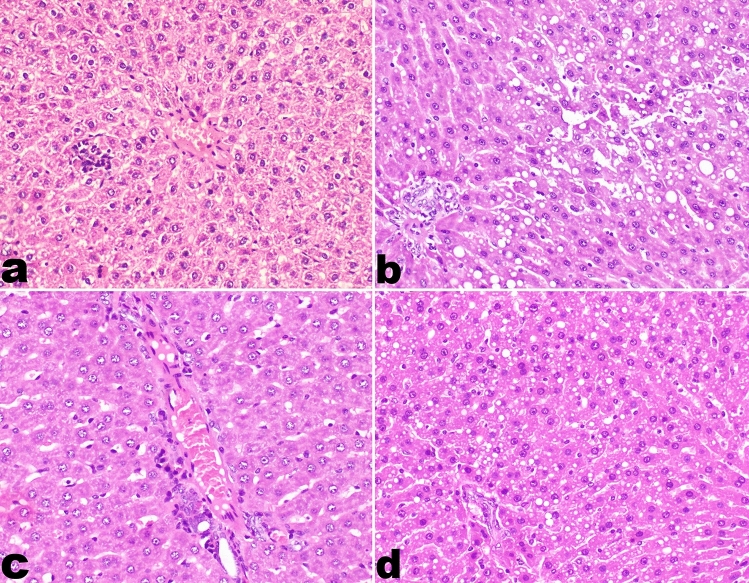
Histopathology of livers of rats. **a** Focal hepatic necrosis with mononuclear inflammatory cells infiltration in group E (Silicate group), **b **diffuse area of macro and micro-hepatic steatosis in group F (Silicate/Prednisolone group), **c** sinusoidal dilatation and periportal inflammatory cells infiltration in group G (Silicate/Myrrh group), **d** area of micro-hepatic steatosis in group H (Silicate/Prednisolone/Myrrh group) (20X) H & E stain

The liver of group B (Prednisolone group) and group F (Silicate/Prednisolone group) treated with prednisolone revealed moderate hepatocellular injury with multifocal to diffuse vacuolar degeneration in addition to the presence of moderate to severe micro-and macro-steatosis after 6 weeks from prednisolone administration in the group (F). The liver of rats in group D (Prednisolone/Myrrh group) and group H (Silicate/Prednisolone/Myrrh group) also showed similar but milder lesions compared to the previously mentioned groups. In group C (Myrrh group) no remarkable changes were observed. On the other hand, the liver in group E (Silicate group) showed dilatation of hepatic sinusoids, sinusoidal leukocytosis and necrotic focus invaded with mononuclear inflammatory cells. In group G (Silicate/Myrrh group), there was mild dilatation of hepatic sinusoids with focal areas of vacuolar degeneration (Fig. 6).

## Discussion

Treatment of an autoimmune disease is still challenging and requires new therapeutic approaches. Induction of autoimmune disease using silica in the present study was confirmed by the presence of a picture of immune-mediated glomerulonephritis and elevated serum urea and creatinine. Similar to previous studies which showed that silica induces glomerulonephritis (Stratta et al. 2001; Zelko et al. 2016), administration of myrrh with or without prednisolone to rats improved renal histopathology, and kidney function, and reduced TNFα with time compared with silica treated group. Prednisolone is a potent anti-inflammatory drug as reported in previous studies which alleviates glomerulonephritis (Paul-Clark et al. 2000; Chafin et al. 2013). The Myrrh, on the other hand, contains guggulsterone a potent anti-inflammatory that causes downregulation of some inflammatory mediators such as TNFα, IFN-γ, IL-1β, or IL-2 and inhibits MAP kinases such as JNK ( c-jun NH2-terminal kinase) pathway and p38 in peripheral blood mononuclear cells (Kim et al. 2012; Su et al. 2015; Rosenthal et al. 2017). Moreover, myrrhanol A (polypodane triterpenoid) inhibits NO (nitric oxide) production which is known to play a central role in inflammatory and immune reactions through inhibition of iNOS (inducible nitric oxide synthase) induction in macrophages (Morikawa et al. 2017). Sesquiterpenoid components such as elema-1,3,11(13)-trien-12-ol, another component of myrrh, also exhibited a potent anti-inflammatory effect and an immunomodulatory effect respectively (Shen et al. 2012; Kim et al. 2013).

In the current study, the leukocytes infiltrated in renal interstitial tissue were completely diminished in the groups treated with prednisolone compared to the group treated with Myrrh. Myrrh has an immunomodulatory effect as mentioned earlier without decreasing the inflammatory cell count (Haffor. 2010), unlike prednisolone which causes severe immunosuppression. Glucocorticoids inhibit vasodilation and the increase in vascular permeability following inflammatory insult to prevent the recruitment of inflammatory cells to sites of inflammation. Furthermore, glucocorticoids promote the cell death of many inflammatory cells which may further contribute to reducing the inflammatory cell burden (Newton. 2000). The use of myrrh and prednisolone together restored the balance in the immune system as observed in the renal lesion score and TNF expression.

Form the drawbacks of long-time prednisolone, administration is hepatic steatosis (Candelli et al. 2003; Chitturi and Farrell. 2013). Similar to our study which also revealed microscopic hepatic steatosis and elevated liver enzymes in the group of rats treated with prednisolone, the administration of myrrh alone to rats did not show the same side effect as prednisolone. This could be due to curzerene (sesquiterpenoid) found in myrrh which possesses antioxidant and free radical neutralizing properties (Zhao et al. 2010; Forman et al. 2014). Moreover, the use of myrrh with prednisolone improved liver histopathology. Consequently, myrrh can be considered a safe natural medicine with the least side effects (Shalaby and Hammouda. 2014).

## Conclusion

Myrrh extract showed an anti-inflammatory effect and subsided the immune-mediated glomerulonephritis. It counteracted the adverse effect of the conventional drug (prednisolone). Myrrh extract is safe and can be used as a conjunctive therapy in the treatment of autoimmune diseases.

## Data Availability

The datasets used during the current study are available from the corresponding author on reasonable request.
